# [Malonato(2−)-κ^2^ *O*,*O*′]bis­(1,10-phenanthroline-κ^2^ *N*,*N*′)zinc(II) penta­hydrate

**DOI:** 10.1107/S1600536810049573

**Published:** 2010-12-11

**Authors:** Yan-Min Chen, Qing-Fan Xie

**Affiliations:** a Department of Chemistry and Science of Life, Quanzhou Normal University, Fujian 362000, People’s Republic of China

## Abstract

In the title complex, [Zn(C_3_H_2_O_4_)(C_12_H_8_N_2_)_2_]·5H_2_O, the Zn^II^ cation displays a distorted octa­hedral geometry, being coordinated by four N atoms from two 1,10-phenanthroline ligands and two O atoms from different carboxyl­ate groups of the chelating malonate dianion. In the crystal, the complexes are linked into a three-dimensional supra­molecular network by both O—H⋯O hydrogen-bonding inter­actions between water mol­ecules and the uncoordinated carboxyl­ate O atoms of neighboring mol­ecules, and aromatic π–π stacking inter­actions between neighboring phenanthroline rings with centroid–centroid distances of 3.4654 (17) and 3.697 (2) Å.

## Related literature

For zinc-aliphatic dicarboxyl­ate complexes with 1,10-phenanthroline as co-ligand, see: Fu *et al.* (2006 ▶); Kuang *et al.* (2007 ▶); Liu *et al.* (2004 ▶); Yang *et al.* (2007 ▶); Zhang *et al.* (2005 ▶); Zheng *et al.* (2002 ▶). For Zn—O and Zn—N bond lengths, see: Guilera & Steed (1999 ▶); Tao *et al.* (2000 ▶).
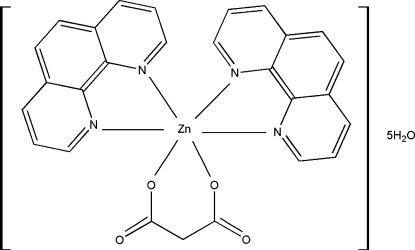


## Experimental

### 

#### Crystal data

[Zn(C_3_H_2_O_4_)(C_12_H_8_N_2_)_2_]·5H_2_O
*M* *_r_* = 617.90
Triclinic, 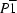

*a* = 10.3802 (15) Å
*b* = 10.580 (3) Å
*c* = 13.059 (2) Å
α = 84.682 (2)°
β = 76.965 (3)°
γ = 72.834 (2)°
*V* = 1334.5 (4) Å^3^
*Z* = 2
Mo *K*α radiation
μ = 0.98 mm^−1^
*T* = 291 K
0.26 × 0.20 × 0.18 mm

#### Data collection

Bruker SMART APEX CCD diffractometer
Absorption correction: multi-scan (*SADABS*; Bruker, 2000 ▶) *T*_min_ = 0.784, *T*_max_ = 0.843
13993 measured reflections
5236 independent reflections
4851 reflections with *I* > 2σ(*I*)
*R*_int_ = 0.045

#### Refinement

*R*[*F* ^2^ > 2σ(*F* ^2^)] = 0.040
*wR*(*F* ^2^) = 0.097
*S* = 1.02
5236 reflections
370 parameters
H-atom parameters constrained
Δρ_max_ = 0.32 e Å^−3^
Δρ_min_ = −0.80 e Å^−3^

### 

Data collection: *SMART* (Bruker, 2000 ▶); cell refinement: *SAINT-Plus* (Bruker, 2000 ▶); data reduction: *SAINT-Plus*; program(s) used to solve structure: *SHELXTL* (Sheldrick, 2008 ▶); program(s) used to refine structure: *SHELXTL*; molecular graphics: *SHELXTL*; software used to prepare material for publication: *SHELXTL*.

## Supplementary Material

Crystal structure: contains datablocks global, I. DOI: 10.1107/S1600536810049573/vm2055sup1.cif

Structure factors: contains datablocks I. DOI: 10.1107/S1600536810049573/vm2055Isup2.hkl

Additional supplementary materials:  crystallographic information; 3D view; checkCIF report

## Figures and Tables

**Table 1:** Hydrogen-bond geometry (Å, °)

*D*—H⋯*A*	*D*—H	H⋯*A*	*D*⋯*A*	*D*—H⋯*A*
O1*W*—H1*X*⋯O2	0.85	1.85	2.696 (3)	178
O1*W*—H1*Y*⋯O5*W*^i^	0.85	2.50	3.085 (3)	127
O2*W*—H2*X*⋯O3*W*^ii^	0.85	2.18	2.836 (4)	133
O2*W*—H2*Y*⋯O4*W*^iii^	0.85	2.09	2.804 (4)	141
O3*W*—H3*X*⋯O1^iv^	0.85	2.21	2.696 (3)	116
O3*W*—H3*Y*⋯O3^v^	0.85	2.27	2.806 (3)	121
O4*W*—H4*X*⋯O3*W*	0.85	2.10	2.953 (3)	180
O4*W*—H4*Y*⋯O1*W*^iv^	0.85	2.07	2.854 (3)	153
O5*W*—H5*X*⋯O1*W*	0.85	1.94	2.789 (3)	179
O5*W*—H5*Y*⋯O2*W*^vi^	0.85	2.21	2.733 (3)	120
O5*W*—H5*Y*⋯O1*W*^i^	0.85	2.57	3.085 (3)	120

Symmetry codes: (i) 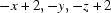; (ii) 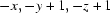; (iii) 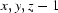; (iv) 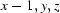; (v) 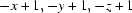; (vi) 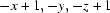.

**Fig. 1.**
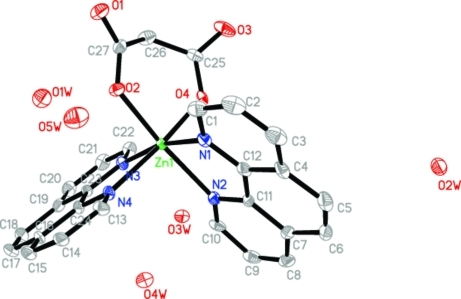
The ORTEP drawing of the title compound (I). Displacement ellipsoids are drawn at 30% probability level. All hydrogen atoms have been omitted for reasons of clarity.

**Fig. 2.**
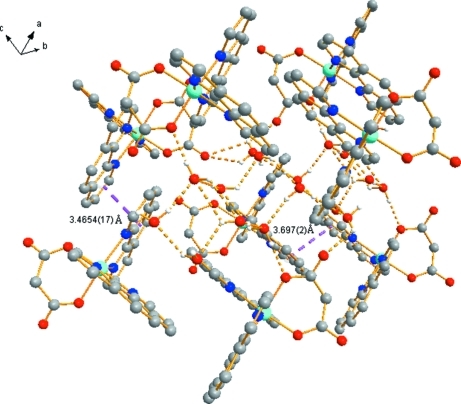
Crystal packing of (I) along bc plane showing H-bonding (yellow dashed lines) and π-π stacking interactions (pink dashed lines).

## supplementary crystallographic information

### Comment

Recently, there have been a number of reports on zinc-aliphatic dicarboxylate complexes with 1,10-phenanthroline (phen) as co-ligand (Fu *et al.* 2006; Kuang *et al.*,2007; Yang *et al.*, 2007; Zheng *et al.*, 2002; Liu *et al.*, 2004; Zhang *et al.*,2005). In order to continue this research, we have synthesized the title complex [Zn(C_3_H_2_O_4_)(C_12_H_8_N_2_)_2_].5H_2_O and have characterized it by elemental analysis and single-crystal X-ray diffraction analysis.

The Zn^II^ cation has a slightly distorted octahedral geometry and is coordinated by four N atoms from two phen ligands and two O atoms from different carboxylate groups of the chelating malonate dianion. The equatorial plane is defined by atoms N1,N2,N3,O2; the apical positions being occupied by N4 and O4. The Zn—O (2.0203–2.0687 (17) Å) and Zn—N (2.141–2.176 (2) Å) bond lengths are in the normal ranges (Guilera *et al.*,1999; Tao, *et al.*, 2000).

The crystal packing is stabilized by intermolecular π-π interactions between the phenyl rings [for example *Cg*(5)···*Cg*(8)^a^ = 3.4654 (17) Å, *Cg*(7)···*Cg*(7)^b^ = 3.697 (2) Å, with *Cg*(5) the centroid of ring N2/C7—C11, *Cg*(8) of ring C4—C12, *Cg*(7) of ring N4/C13-C16/C24, symmetry codes: (a) 1 - *x*,-*y*, 1 - *z*, (b) 1 - *x*,-*y*, 2 - *z*], and O—H···O hydrogen bonds (Table 1, Fig. 2).

### Experimental

An ethanol solution (10 ml) of 1,10-phenanthroline (0.397 g, 2 mmol), an aqueous solution (10 ml) of Zn(NO_3_)_2_.6H_2_O (0.297 g, 1 mmol) and an aqueous solution (10 ml) of malonic acid (0.104 g, 1 mmol) were mixed. After refluxing for 4 h, the hot mixture was filtered off. The colorless single crystals suitable for X-ray analysis were obtained by slow evaporation of the filtrate at room temperature after 7 days.

### Refinement

H atoms bonded to C were placed geometrically and treated as riding, (C—H = 0.93–0.97 Å), with *U*_iso_(H) = 1.2*U*_eq_(C). The water H atoms found from Fourier difference maps were refined with restraints for O—H distances (0.8500 Å) and and *U*_iso_(H) = 1.5*U*_eq_(O).

### Crystal data

**Table:**

[Zn(C_3_H_2_O_4_)(C_12_H_8_N_2_)_2_]·5H_2_O	*Z* = 2
*M**_r_* = 617.90	*F*(000) = 640
Triclinic, *P*1	*D*_x_ = 1.538 Mg m^−^^3^
Hall symbol: -P 1	Mo *K*α radiation, λ = 0.71073 Å
*a* = 10.3802 (15) Å	Cell parameters from 6521 reflections
*b* = 10.580 (3) Å	θ = 2.5–28.0°
*c* = 13.059 (2) Å	µ = 0.98 mm^−^^1^
α = 84.682 (2)°	*T* = 291 K
β = 76.965 (3)°	Bolck, colorless
γ = 72.834 (2)°	0.26 × 0.20 × 0.18 mm
*V* = 1334.5 (4) Å^3^	

### Data collection

**Table:**

Bruker SMART APEX CCD diffractometer	5236 independent reflections
Radiation source: sealed tube	4851 reflections with *I* > 2σ(*I*)
graphite	*R*_int_ = 0.045
phi and ω scans	θ_max_ = 26.0°, θ_min_ = 1.6°
Absorption correction: multi-scan (*SADABS*; Bruker, 2000)	*h* = −12→12
*T*_min_ = 0.784, *T*_max_ = 0.843	*k* = −13→13
13993 measured reflections	*l* = −16→16

### Refinement

**Table:**

Refinement on *F*^2^	Primary atom site location: structure-invariant direct methods
Least-squares matrix: full	Secondary atom site location: difference Fourier map
*R*[*F*^2^ > 2σ(*F*^2^)] = 0.040	Hydrogen site location: inferred from neighbouring sites
*wR*(*F*^2^) = 0.097	H-atom parameters constrained
*S* = 1.02	*w* = 1/[σ^2^(*F*_o_^2^) + (0.05*P*)^2^ + 1.22*P*] where *P* = (*F*_o_^2^ + 2*F*_c_^2^)/3
5236 reflections	(Δ/σ)_max_ < 0.001
370 parameters	Δρ_max_ = 0.32 e Å^−^^3^
0 restraints	Δρ_min_ = −0.80 e Å^−^^3^

### Special details

**Table:**

Geometry. All e.s.d.'s (except the e.s.d. in the dihedral angle between two l.s. planes) are estimated using the full covariance matrix. The cell e.s.d.'s are taken into account individually in the estimation of e.s.d.'s in distances, angles and torsion angles; correlations between e.s.d.'s in cell parameters are only used when they are defined by crystal symmetry. An approximate (isotropic) treatment of cell e.s.d.'s is used for estimating e.s.d.'s involving l.s. planes.
Refinement. Refinement of *F*^2^ against ALL reflections. The weighted *R*-factor *wR* and goodness of fit *S* are based on *F*^2^, conventional *R*-factors *R* are based on *F*, with *F* set to zero for negative *F*^2^. The threshold expression of *F*^2^ > σ(*F*^2^) is used only for calculating *R*-factors(gt) *etc*. and is not relevant to the choice of reflections for refinement. *R*-factors based on *F*^2^ are statistically about twice as large as those based on *F*, and *R*- factors based on ALL data will be even larger.

### Fractional atomic coordinates and isotropic or equivalent isotropic displacement parameters (Å^2^)

**Table:**

	*x*	*y*	*z*	*U*_iso_*/*U*_eq_	
C1	0.8893 (3)	−0.0210 (3)	0.6477 (2)	0.0372 (6)	
H1A	0.9374	0.0280	0.6714	0.045*	
C2	0.9626 (3)	−0.1418 (3)	0.5995 (3)	0.0445 (8)	
H2A	1.0580	−0.1720	0.5906	0.053*	
C3	0.8934 (3)	−0.2128 (3)	0.5667 (3)	0.0449 (8)	
H3A	0.9417	−0.2926	0.5343	0.054*	
C4	0.7496 (3)	−0.1694 (2)	0.5801 (2)	0.0316 (6)	
C5	0.6684 (4)	−0.2373 (3)	0.5447 (2)	0.0427 (8)	
H5A	0.7120	−0.3134	0.5062	0.051*	
C6	0.5299 (4)	−0.1932 (3)	0.5660 (2)	0.0384 (7)	
H6A	0.4798	−0.2431	0.5461	0.046*	
C7	0.4589 (3)	−0.0738 (3)	0.61756 (19)	0.0291 (6)	
C8	0.3162 (3)	−0.0214 (3)	0.6421 (2)	0.0396 (7)	
H8A	0.2604	−0.0678	0.6259	0.048*	
C9	0.2576 (3)	0.0969 (3)	0.6895 (2)	0.0379 (7)	
H9A	0.1622	0.1309	0.7064	0.045*	
C10	0.3413 (3)	0.1664 (3)	0.7123 (2)	0.0321 (6)	
H10A	0.3002	0.2489	0.7422	0.038*	
C11	0.5355 (3)	0.0041 (2)	0.64652 (17)	0.0228 (5)	
C12	0.6836 (3)	−0.0471 (2)	0.62902 (19)	0.0268 (5)	
C13	0.6790 (3)	0.0354 (3)	0.9213 (2)	0.0289 (5)	
H13A	0.7381	−0.0279	0.8743	0.035*	
C14	0.6684 (3)	0.0069 (3)	1.0273 (2)	0.0302 (6)	
H14A	0.7184	−0.0742	1.0514	0.036*	
C15	0.5817 (3)	0.1018 (3)	1.0975 (2)	0.0349 (6)	
H15A	0.5760	0.0869	1.1695	0.042*	
C16	0.5016 (3)	0.2219 (3)	1.05814 (18)	0.0266 (5)	
C17	0.4076 (3)	0.3224 (3)	1.1248 (2)	0.0366 (6)	
H17A	0.3955	0.3090	1.1974	0.044*	
C18	0.3351 (3)	0.4380 (3)	1.0847 (2)	0.0373 (6)	
H18A	0.2722	0.5014	1.1299	0.045*	
C19	0.3548 (3)	0.4622 (3)	0.9741 (2)	0.0281 (5)	
C20	0.2867 (3)	0.5812 (3)	0.9265 (2)	0.0352 (6)	
H20A	0.2241	0.6489	0.9678	0.042*	
C21	0.3131 (3)	0.5961 (3)	0.8210 (2)	0.0350 (6)	
H21A	0.2692	0.6745	0.7893	0.042*	
C22	0.4062 (3)	0.4940 (3)	0.7592 (2)	0.0288 (5)	
H22A	0.4223	0.5052	0.6864	0.035*	
C23	0.4500 (2)	0.3641 (2)	0.90571 (18)	0.0205 (5)	
C24	0.5217 (2)	0.2419 (2)	0.94922 (18)	0.0225 (5)	
C25	0.7182 (2)	0.3692 (3)	0.5295 (2)	0.0279 (5)	
C26	0.7806 (3)	0.4298 (3)	0.5965 (2)	0.0340 (6)	
H26A	0.7076	0.4980	0.6369	0.041*	
H26B	0.8433	0.4731	0.5507	0.041*	
C27	0.8586 (3)	0.3378 (2)	0.6726 (2)	0.0272 (5)	
N1	0.7535 (2)	0.0252 (2)	0.66042 (16)	0.0263 (4)	
N2	0.4776 (2)	0.1201 (2)	0.69320 (15)	0.0241 (4)	
N3	0.4729 (2)	0.3804 (2)	0.80156 (15)	0.0223 (4)	
N4	0.6074 (2)	0.1510 (2)	0.88198 (15)	0.0229 (4)	
O1	0.9787 (2)	0.3391 (2)	0.67131 (18)	0.0408 (5)	
O2	0.79835 (18)	0.26528 (18)	0.73556 (15)	0.0295 (4)	
O3	0.7436 (2)	0.3892 (2)	0.43388 (16)	0.0487 (6)	
O4	0.6369 (2)	0.30087 (19)	0.57407 (13)	0.0314 (4)	
O1W	0.9384 (2)	0.16928 (19)	0.88973 (17)	0.0396 (5)	
H1X	0.8963	0.2004	0.8401	0.048*	
H1Y	0.8802	0.1636	0.9460	0.048*	
O2W	0.0797 (3)	0.2733 (3)	0.12852 (19)	0.0545 (6)	
H2X	0.0151	0.3453	0.1366	0.065*	
H2Y	0.1286	0.2722	0.0667	0.065*	
O3W	0.0798 (2)	0.5202 (2)	0.73284 (17)	0.0471 (5)	
H3X	0.0796	0.4957	0.6727	0.056*	
H3Y	0.1389	0.5629	0.7266	0.056*	
O4W	0.1087 (3)	0.3290 (2)	0.91176 (19)	0.0523 (6)	
H4X	0.1009	0.3840	0.8602	0.063*	
H4Y	0.0423	0.2945	0.9240	0.063*	
O5W	1.0707 (2)	−0.1012 (2)	0.8743 (2)	0.0508 (6)	
H5X	1.0299	−0.0188	0.8783	0.061*	
H5Y	1.0258	−0.1433	0.9203	0.061*	
Zn1	0.62469 (3)	0.21697 (3)	0.720044 (19)	0.01839 (9)	

### Atomic displacement parameters (Å^2^)

**Table:**

	*U*^11^	*U*^22^	*U*^33^	*U*^12^	*U*^13^	*U*^23^
C1	0.0354 (15)	0.0360 (15)	0.0394 (16)	−0.0120 (12)	−0.0069 (12)	0.0069 (12)
C2	0.0302 (15)	0.0357 (16)	0.0507 (19)	0.0058 (12)	0.0034 (13)	0.0042 (14)
C3	0.0472 (18)	0.0308 (15)	0.0412 (17)	−0.0010 (13)	0.0099 (14)	−0.0046 (12)
C4	0.0503 (16)	0.0192 (12)	0.0188 (12)	−0.0034 (11)	−0.0040 (11)	0.0031 (9)
C5	0.083 (2)	0.0234 (13)	0.0221 (13)	−0.0156 (15)	−0.0127 (14)	0.0061 (10)
C6	0.077 (2)	0.0307 (14)	0.0188 (12)	−0.0303 (15)	−0.0168 (13)	0.0102 (10)
C7	0.0485 (16)	0.0269 (13)	0.0206 (12)	−0.0204 (12)	−0.0176 (11)	0.0137 (10)
C8	0.0504 (18)	0.0525 (18)	0.0298 (14)	−0.0336 (15)	−0.0190 (13)	0.0195 (13)
C9	0.0294 (14)	0.0559 (19)	0.0283 (14)	−0.0158 (13)	−0.0087 (11)	0.0172 (13)
C10	0.0282 (13)	0.0441 (16)	0.0210 (12)	−0.0111 (12)	−0.0045 (10)	0.0145 (11)
C11	0.0324 (13)	0.0266 (12)	0.0136 (11)	−0.0132 (10)	−0.0097 (9)	0.0068 (9)
C12	0.0384 (14)	0.0242 (12)	0.0170 (11)	−0.0093 (10)	−0.0065 (10)	0.0071 (9)
C13	0.0258 (12)	0.0339 (14)	0.0271 (13)	−0.0084 (11)	−0.0108 (10)	0.0115 (10)
C14	0.0349 (14)	0.0361 (14)	0.0249 (13)	−0.0172 (11)	−0.0133 (11)	0.0143 (11)
C15	0.0445 (16)	0.0447 (16)	0.0215 (13)	−0.0233 (13)	−0.0109 (11)	0.0137 (11)
C16	0.0360 (14)	0.0366 (14)	0.0126 (11)	−0.0192 (11)	−0.0053 (10)	0.0024 (9)
C17	0.0388 (15)	0.0521 (18)	0.0205 (13)	−0.0226 (14)	0.0028 (11)	0.0009 (12)
C18	0.0362 (15)	0.0514 (18)	0.0255 (14)	−0.0190 (13)	0.0031 (11)	−0.0096 (12)
C19	0.0230 (12)	0.0322 (13)	0.0316 (14)	−0.0103 (10)	−0.0042 (10)	−0.0090 (11)
C20	0.0300 (14)	0.0326 (14)	0.0407 (16)	−0.0055 (11)	−0.0020 (12)	−0.0141 (12)
C21	0.0273 (13)	0.0298 (14)	0.0460 (17)	0.0000 (11)	−0.0125 (12)	−0.0057 (12)
C22	0.0221 (12)	0.0271 (13)	0.0361 (14)	0.0009 (10)	−0.0151 (10)	0.0016 (11)
C23	0.0204 (11)	0.0206 (11)	0.0204 (11)	−0.0065 (9)	−0.0009 (9)	−0.0048 (9)
C24	0.0243 (12)	0.0290 (12)	0.0168 (11)	−0.0092 (10)	−0.0076 (9)	0.0005 (9)
C25	0.0200 (11)	0.0294 (13)	0.0251 (13)	0.0009 (10)	−0.0003 (9)	0.0082 (10)
C26	0.0308 (14)	0.0235 (13)	0.0468 (17)	−0.0074 (11)	−0.0114 (12)	0.0097 (11)
C27	0.0264 (13)	0.0245 (12)	0.0323 (14)	−0.0081 (10)	−0.0067 (10)	−0.0044 (10)
N1	0.0288 (11)	0.0261 (11)	0.0213 (10)	−0.0063 (9)	−0.0011 (8)	−0.0016 (8)
N2	0.0294 (11)	0.0265 (10)	0.0186 (10)	−0.0094 (9)	−0.0083 (8)	0.0022 (8)
N3	0.0238 (10)	0.0236 (10)	0.0188 (10)	−0.0035 (8)	−0.0090 (8)	0.0031 (8)
N4	0.0243 (10)	0.0279 (11)	0.0184 (10)	−0.0070 (8)	−0.0096 (8)	0.0022 (8)
O1	0.0347 (11)	0.0441 (12)	0.0501 (13)	−0.0188 (9)	−0.0134 (9)	0.0028 (10)
O2	0.0279 (9)	0.0321 (10)	0.0327 (10)	−0.0140 (8)	−0.0112 (8)	0.0077 (8)
O3	0.0364 (11)	0.0706 (15)	0.0292 (11)	−0.0089 (11)	−0.0051 (9)	0.0235 (10)
O4	0.0441 (11)	0.0458 (11)	0.0104 (8)	−0.0196 (9)	−0.0109 (7)	0.0050 (7)
O1W	0.0443 (12)	0.0335 (11)	0.0426 (12)	−0.0116 (9)	−0.0145 (9)	0.0064 (9)
O2W	0.0491 (14)	0.0714 (17)	0.0434 (13)	−0.0173 (12)	−0.0070 (11)	−0.0093 (12)
O3W	0.0569 (14)	0.0598 (14)	0.0345 (11)	−0.0361 (12)	−0.0054 (10)	0.0036 (10)
O4W	0.0572 (14)	0.0594 (15)	0.0488 (14)	−0.0324 (12)	−0.0072 (11)	−0.0004 (11)
O5W	0.0500 (13)	0.0340 (11)	0.0665 (16)	−0.0121 (10)	−0.0082 (11)	−0.0007 (11)
Zn1	0.02194 (15)	0.02086 (15)	0.01283 (14)	−0.00678 (10)	−0.00444 (10)	0.00161 (9)

### Geometric parameters (Å, °)

**Table:**

C1—N1	1.326 (4)	C18—H18A	0.9300
C1—C2	1.403 (4)	C19—C20	1.414 (4)
C1—H1A	0.9300	C19—C23	1.422 (3)
C2—C3	1.334 (5)	C20—C21	1.347 (4)
C2—H2A	0.9300	C20—H20A	0.9300
C3—C4	1.402 (4)	C21—C22	1.395 (4)
C3—H3A	0.9300	C21—H21A	0.9300
C4—C12	1.409 (4)	C22—N3	1.337 (3)
C4—C5	1.426 (4)	C22—H22A	0.9300
C5—C6	1.345 (5)	C23—N3	1.331 (3)
C5—H5A	0.9300	C23—C24	1.427 (3)
C6—C7	1.406 (4)	C24—N4	1.336 (3)
C6—H6A	0.9300	C25—O3	1.228 (3)
C7—C8	1.392 (4)	C25—O4	1.278 (3)
C7—C11	1.427 (3)	C25—C26	1.483 (4)
C8—C9	1.357 (5)	C26—C27	1.520 (4)
C8—H8A	0.9300	C26—H26A	0.9700
C9—C10	1.385 (4)	C26—H26B	0.9700
C9—H9A	0.9300	C27—O1	1.247 (3)
C10—N2	1.327 (3)	C27—O2	1.261 (3)
C10—H10A	0.9300	N1—Zn1	2.175 (2)
C11—N2	1.333 (3)	N2—Zn1	2.176 (2)
C11—C12	1.444 (4)	N3—Zn1	2.141 (2)
C12—N1	1.341 (3)	N4—Zn1	2.150 (2)
C13—N4	1.353 (3)	O2—Zn1	2.0687 (18)
C13—C14	1.376 (4)	O4—Zn1	2.0203 (17)
C13—H13A	0.9300	O1W—H1X	0.8500
C14—C15	1.387 (4)	O1W—H1Y	0.8500
C14—H14A	0.9300	O2W—H2X	0.8500
C15—C16	1.420 (4)	O2W—H2Y	0.8501
C15—H15A	0.9300	O3W—H3X	0.8500
C16—C24	1.396 (3)	O3W—H3Y	0.8500
C16—C17	1.420 (4)	O4W—H4X	0.8500
C17—C18	1.361 (4)	O4W—H4Y	0.8500
C17—H17A	0.9300	O5W—H5X	0.8500
C18—C19	1.423 (4)	O5W—H5Y	0.8501
			
N1—C1—C2	121.8 (3)	C19—C20—H20A	120.2
N1—C1—H1A	119.1	C20—C21—C22	120.0 (3)
C2—C1—H1A	119.1	C20—C21—H21A	120.0
C3—C2—C1	119.1 (3)	C22—C21—H21A	120.0
C3—C2—H2A	120.5	N3—C22—C21	121.9 (3)
C1—C2—H2A	120.5	N3—C22—H22A	119.0
C2—C3—C4	121.3 (3)	C21—C22—H22A	119.0
C2—C3—H3A	119.3	N3—C23—C19	122.4 (2)
C4—C3—H3A	119.3	N3—C23—C24	118.2 (2)
C3—C4—C12	116.2 (3)	C19—C23—C24	119.4 (2)
C3—C4—C5	124.8 (3)	N4—C24—C16	122.8 (2)
C12—C4—C5	119.0 (3)	N4—C24—C23	117.4 (2)
C6—C5—C4	121.2 (3)	C16—C24—C23	119.8 (2)
C6—C5—H5A	119.4	O3—C25—O4	122.5 (3)
C4—C5—H5A	119.4	O3—C25—C26	119.0 (2)
C5—C6—C7	121.8 (3)	O4—C25—C26	118.5 (2)
C5—C6—H6A	119.1	C25—C26—C27	117.0 (2)
C7—C6—H6A	119.1	C25—C26—H26A	108.0
C8—C7—C6	125.3 (3)	C27—C26—H26A	108.0
C8—C7—C11	115.5 (3)	C25—C26—H26B	108.0
C6—C7—C11	119.3 (3)	C27—C26—H26B	108.0
C9—C8—C7	120.7 (3)	H26A—C26—H26B	107.3
C9—C8—H8A	119.6	O1—C27—O2	122.8 (3)
C7—C8—H8A	119.6	O1—C27—C26	118.5 (2)
C8—C9—C10	119.4 (3)	O2—C27—C26	118.6 (2)
C8—C9—H9A	120.3	C1—N1—C12	119.2 (2)
C10—C9—H9A	120.3	C1—N1—Zn1	127.06 (19)
N2—C10—C9	122.7 (3)	C12—N1—Zn1	113.63 (17)
N2—C10—H10A	118.7	C10—N2—C11	118.1 (2)
C9—C10—H10A	118.7	C10—N2—Zn1	127.68 (19)
N2—C11—C7	123.7 (2)	C11—N2—Zn1	114.13 (16)
N2—C11—C12	117.5 (2)	C23—N3—C22	119.2 (2)
C7—C11—C12	118.7 (2)	C23—N3—Zn1	113.65 (15)
N1—C12—C4	122.4 (3)	C22—N3—Zn1	127.12 (18)
N1—C12—C11	117.9 (2)	C24—N4—C13	118.5 (2)
C4—C12—C11	119.6 (2)	C24—N4—Zn1	113.60 (16)
N4—C13—C14	123.2 (3)	C13—N4—Zn1	127.68 (17)
N4—C13—H13A	118.4	C27—O2—Zn1	125.77 (17)
C14—C13—H13A	118.4	C25—O4—Zn1	126.93 (16)
C13—C14—C15	118.5 (2)	H1X—O1W—H1Y	109.5
C13—C14—H14A	120.7	H2X—O2W—H2Y	109.5
C15—C14—H14A	120.7	H3X—O3W—H3Y	109.5
C14—C15—C16	119.2 (2)	H4X—O4W—H4Y	109.5
C14—C15—H15A	120.4	H5X—O5W—H5Y	109.5
C16—C15—H15A	120.4	O4—Zn1—O2	90.76 (7)
C24—C16—C17	119.7 (2)	O4—Zn1—N3	97.33 (8)
C24—C16—C15	117.6 (2)	O2—Zn1—N3	97.98 (8)
C17—C16—C15	122.7 (2)	O4—Zn1—N4	173.14 (8)
C18—C17—C16	121.3 (2)	O2—Zn1—N4	86.27 (7)
C18—C17—H17A	119.3	N3—Zn1—N4	76.99 (8)
C16—C17—H17A	119.3	O4—Zn1—N1	92.29 (8)
C17—C18—C19	120.4 (3)	O2—Zn1—N1	89.90 (8)
C17—C18—H18A	119.8	N3—Zn1—N1	167.45 (8)
C19—C18—H18A	119.8	N4—Zn1—N1	93.89 (8)
C20—C19—C23	116.8 (2)	O4—Zn1—N2	90.97 (7)
C20—C19—C18	123.8 (3)	O2—Zn1—N2	166.13 (8)
C23—C19—C18	119.4 (2)	N3—Zn1—N2	95.45 (8)
C21—C20—C19	119.7 (2)	N4—Zn1—N2	93.38 (8)
C21—C20—H20A	120.2	N1—Zn1—N2	76.28 (8)
			
N1—C1—C2—C3	−0.8 (5)	C12—C11—N2—Zn1	5.5 (3)
C1—C2—C3—C4	−0.4 (5)	C19—C23—N3—C22	1.3 (4)
C2—C3—C4—C12	0.5 (4)	C24—C23—N3—C22	179.6 (2)
C2—C3—C4—C5	178.1 (3)	C19—C23—N3—Zn1	178.89 (18)
C3—C4—C5—C6	176.1 (3)	C24—C23—N3—Zn1	−2.8 (3)
C12—C4—C5—C6	−6.4 (4)	C21—C22—N3—C23	0.0 (4)
C4—C5—C6—C7	4.5 (4)	C21—C22—N3—Zn1	−177.25 (19)
C5—C6—C7—C8	179.8 (3)	C16—C24—N4—C13	−0.8 (4)
C5—C6—C7—C11	1.1 (4)	C23—C24—N4—C13	178.6 (2)
C6—C7—C8—C9	−178.1 (2)	C16—C24—N4—Zn1	−176.35 (19)
C11—C7—C8—C9	0.6 (4)	C23—C24—N4—Zn1	3.0 (3)
C7—C8—C9—C10	0.8 (4)	C14—C13—N4—C24	−0.4 (4)
C8—C9—C10—N2	−2.5 (4)	C14—C13—N4—Zn1	174.43 (19)
C8—C7—C11—N2	−0.5 (3)	O1—C27—O2—Zn1	−162.6 (2)
C6—C7—C11—N2	178.3 (2)	C26—C27—O2—Zn1	18.8 (3)
C8—C7—C11—C12	176.6 (2)	O3—C25—O4—Zn1	161.7 (2)
C6—C7—C11—C12	−4.6 (3)	C26—C25—O4—Zn1	−20.2 (3)
C3—C4—C12—N1	0.6 (4)	C25—O4—Zn1—O2	−8.0 (2)
C5—C4—C12—N1	−177.1 (2)	C25—O4—Zn1—N3	90.2 (2)
C3—C4—C12—C11	−179.5 (2)	C25—O4—Zn1—N1	−97.9 (2)
C5—C4—C12—C11	2.8 (3)	C25—O4—Zn1—N2	−174.2 (2)
N2—C11—C12—N1	−0.2 (3)	C27—O2—Zn1—O4	8.4 (2)
C7—C11—C12—N1	−177.5 (2)	C27—O2—Zn1—N3	−89.1 (2)
N2—C11—C12—C4	179.9 (2)	C27—O2—Zn1—N4	−165.4 (2)
C7—C11—C12—C4	2.6 (3)	C27—O2—Zn1—N1	100.7 (2)
N4—C13—C14—C15	−0.8 (4)	C27—O2—Zn1—N2	105.6 (3)
C13—C14—C15—C16	3.2 (4)	C23—N3—Zn1—O4	−172.87 (16)
C14—C15—C16—C24	−4.2 (4)	C22—N3—Zn1—O4	4.5 (2)
C14—C15—C16—C17	178.3 (3)	C23—N3—Zn1—O2	−81.06 (17)
C24—C16—C17—C18	0.8 (4)	C22—N3—Zn1—O2	96.3 (2)
C15—C16—C17—C18	178.2 (3)	C23—N3—Zn1—N4	3.22 (16)
C16—C17—C18—C19	−1.8 (4)	C22—N3—Zn1—N4	−179.4 (2)
C17—C18—C19—C20	−177.9 (3)	C23—N3—Zn1—N1	47.4 (4)
C17—C18—C19—C23	0.6 (4)	C22—N3—Zn1—N1	−135.2 (3)
C23—C19—C20—C21	0.6 (4)	C23—N3—Zn1—N2	95.44 (17)
C18—C19—C20—C21	179.2 (3)	C22—N3—Zn1—N2	−87.2 (2)
C19—C20—C21—C22	0.6 (4)	C24—N4—Zn1—O2	95.76 (17)
C20—C21—C22—N3	−1.0 (4)	C13—N4—Zn1—O2	−79.3 (2)
C20—C19—C23—N3	−1.6 (4)	C24—N4—Zn1—N3	−3.32 (16)
C18—C19—C23—N3	179.8 (2)	C13—N4—Zn1—N3	−178.4 (2)
C20—C19—C23—C24	−179.9 (2)	C24—N4—Zn1—N1	−174.59 (17)
C18—C19—C23—C24	1.4 (4)	C13—N4—Zn1—N1	10.4 (2)
C17—C16—C24—N4	−179.3 (2)	C24—N4—Zn1—N2	−98.13 (17)
C15—C16—C24—N4	3.1 (4)	C13—N4—Zn1—N2	86.8 (2)
C17—C16—C24—C23	1.3 (4)	C1—N1—Zn1—O4	91.7 (2)
C15—C16—C24—C23	−176.2 (2)	C12—N1—Zn1—O4	−84.51 (17)
N3—C23—C24—N4	−0.2 (3)	C1—N1—Zn1—O2	0.9 (2)
C19—C23—C24—N4	178.2 (2)	C12—N1—Zn1—O2	−175.27 (17)
N3—C23—C24—C16	179.2 (2)	C1—N1—Zn1—N3	−128.2 (4)
C19—C23—C24—C16	−2.4 (3)	C12—N1—Zn1—N3	55.6 (4)
O3—C25—C26—C27	−127.4 (3)	C1—N1—Zn1—N4	−85.4 (2)
O4—C25—C26—C27	54.4 (3)	C12—N1—Zn1—N4	98.47 (17)
C25—C26—C27—O1	127.6 (3)	C1—N1—Zn1—N2	−177.9 (2)
C25—C26—C27—O2	−53.7 (4)	C12—N1—Zn1—N2	5.93 (16)
C2—C1—N1—C12	1.9 (4)	C10—N2—Zn1—O4	−89.9 (2)
C2—C1—N1—Zn1	−174.1 (2)	C11—N2—Zn1—O4	86.02 (16)
C4—C12—N1—C1	−1.8 (4)	C10—N2—Zn1—O2	173.0 (3)
C11—C12—N1—C1	178.3 (2)	C11—N2—Zn1—O2	−11.1 (4)
C4—C12—N1—Zn1	174.74 (18)	C10—N2—Zn1—N3	7.6 (2)
C11—C12—N1—Zn1	−5.2 (3)	C11—N2—Zn1—N3	−176.52 (16)
C9—C10—N2—C11	2.6 (3)	C10—N2—Zn1—N4	84.9 (2)
C9—C10—N2—Zn1	178.34 (18)	C11—N2—Zn1—N4	−99.28 (16)
C7—C11—N2—C10	−1.1 (3)	C10—N2—Zn1—N1	178.0 (2)
C12—C11—N2—C10	−178.2 (2)	C11—N2—Zn1—N1	−6.10 (15)
C7—C11—N2—Zn1	−177.38 (17)		

### Hydrogen-bond geometry (Å, °)

**Table:**

*D*—H···*A*	*D*—H	H···*A*	*D*···*A*	*D*—H···*A*
O1W—H1X···O2	0.85	1.85	2.696 (3)	178
O1W—H1Y···O5W^i^	0.85	2.50	3.085 (3)	127
O2W—H2X···O3W^ii^	0.85	2.18	2.836 (4)	133
O2W—H2Y···O4W^iii^	0.85	2.09	2.804 (4)	141
O3W—H3X···O1^iv^	0.85	2.21	2.696 (3)	116
O3W—H3Y···O3^v^	0.85	2.27	2.806 (3)	121
O4W—H4X···O3W	0.85	2.10	2.953 (3)	180
O4W—H4Y···O1W^iv^	0.85	2.07	2.854 (3)	153
O5W—H5X···O1W	0.85	1.94	2.789 (3)	179
O5W—H5Y···O2W^vi^	0.85	2.21	2.733 (3)	120
O5W—H5Y···O1W^i^	0.85	2.57	3.085 (3)	120

Symmetry codes: (i) −*x*+2, −*y*, −*z*+2; (ii) −*x*, −*y*+1, −*z*+1; (iii) *x*, *y*, *z*−1; (iv) *x*−1, *y*, *z*; (v) −*x*+1, −*y*+1, −*z*+1; (vi) −*x*+1, −*y*, −*z*+1.

## References

[bb1] Bruker (2000). *SMART*, *SAINT-Plus* and *SADABS* Bruker AXS Inc., Madison, Wisconsin, USA.

[bb2] Fu, X.-C., Li, M.-T., Wang, C.-G. & Wang, X.-Y. (2006). *Acta Cryst.* C**62**, m13–m15. 10.1107/S0108270105038047 16397329

[bb3] Guilera, G. & Steed, J. W. (1999). *Chem. Commun.* pp. 1563–1564.

[bb4] Kuang, Y. F., Li, C. H., Yang, Y. Q. & Li, W. (2007). *Chin. J. Inorg. Chem.* **23**, 541–544.

[bb5] Liu, Q., Li, Y. Z., Song, Y., Liu, H. J. & Xu, Z. (2004). *J.Solid. State. Chem.* **177**, 4701–4705.

[bb6] Sheldrick, G. M. (2008). *Acta Cryst.* A**64**, 112–122. 10.1107/S0108767307043930 18156677

[bb7] Tao, J., Tong, M. L. & Chen, X. M. (2000). *J. Chem. Soc. Dalton Trans.* pp. 3669–3674.

[bb8] Yang, H., Han, X. L., Zhang, Z. H. & Jiang, P. (2007). *Chin. J. Inorg. Chem.* **23**, 513–516.

[bb9] Zhang, Q. Z., Yang, W. B., Chen, S. M. & Lu, C. Z. (2005). *Bull. Korean Chem. Soc.* **26**, 1631–1634.

[bb10] Zheng, Y. Q., Liu, W. H. & Lin, J. L. (2002). *Z. Anorg. Allg. Chem.* **628**, 620–624.

